# Edible wild mushrooms of the Western Ghats: Data on the ethnic knowledge

**DOI:** 10.1016/j.dib.2017.07.067

**Published:** 2017-07-27

**Authors:** Namera C. Karun, Kandikere R. Sridhar

**Affiliations:** Department of Biosciences, Mangalore University, Mangalagangotri, Mangalore 574199, Karnataka, India

**Keywords:** Macrofungi, Forests, Tree species, Substrate, Mutualistic association, Conservation, Ethnic population, Tribes, Food security, Nutraceuticals, Traditional knowledge

## Abstract

The edible wild mushrooms are most important in food security of ethnic groups and tribals throughout the world. Various indigenous strategies are followed to trace wild mushrooms suitable for human consumption. Data presented in this article projects ethnic knowledge on 51 edible wild mushrooms (in 23 genera) in the Western Ghats region of India. Information collected with support of ethnic groups/tribals pertains to habitats, substrates, mutualistic association, extent of availability, extent of edibility and method of processing of wild mushrooms. Extensive field visits and interactions with ethnic groups were performed to collect the data on each mushroom. Initially, most of these mushrooms were identified based on the indigenous methods and designated with vernacular names (Are-Gowda, Kodava and Tulu). Based on macromorphology (in field) and micromorphology (in laboratory), each mushroom was identified with its systematic name. Among the 51 wild mushrooms irrespective of extent of availability, the most preferred include *Astraeus hygrometricus, Clitocybe infundibuliformis, Fistulina hepatica, Lentinus sajor-caju, Pleurotus* (5 spp.) and *Scleroderma citrinum* and *Termitomyces* (18 spp.). This data forecasts the importance of documentation of traditional knowledge, protection of habitats, management of resources (tree species and substrates) and sustainable exploitation of wild mushrooms.

**Specifications Table**

**Table:** 

Subject area	*Biology*
More specific subject area	*Mycology*
Type of data	*Tables, graphs, and pictures*
How data was acquired	*Interviews, survey, field monitoring (macromorphology), laboratory studies (micromorphology), and photographs*
Data format	*Filtered, and analyzed*
Data source location	*Western Ghats region of India*
Data accessibility	http://shodhganga.inflibnet.ac.in/handle/10603/131845

**Value of data**

• The data presented here project ethnic knowledge on 51 edible wild mushrooms occurring in different regions of the Western Ghats of India.
• Without overlap, wood/monocot stub and soil consists of 18 (12 genera) and 33 (11 genera) edible mushrooms, respectively indicating their substrate specificity.
• The data project the importance of geographic location, substrate and ecological conditions suitable for growth of edible mushrooms, which is of immense value for conservation.
• There is a great variation in the quality within the substrate preferred by mushrooms and likely this factor influences the extent of occurrence (biomass) and nutritional attributes.
• The data on type of mushroom, ethnic name, habitats, substrates, extent of edibility, extent of availability (biomass), mutualistic associations (ectomycorrhizal and termites) and their nutritional values stimulates further research.
• Owing to nutritional and nutraceutical significance of edible mushrooms, our data forecasts strategies for restoration/conservation of habitats and possibilities of *ex situ* cultivation as measure of food security and health of tribals.
• This data also forecast the value of edible wild mushrooms especially geographic indications and intellectual property rights for indigenous knowledge and food security.

## 1 Data

**Fig. 1:**
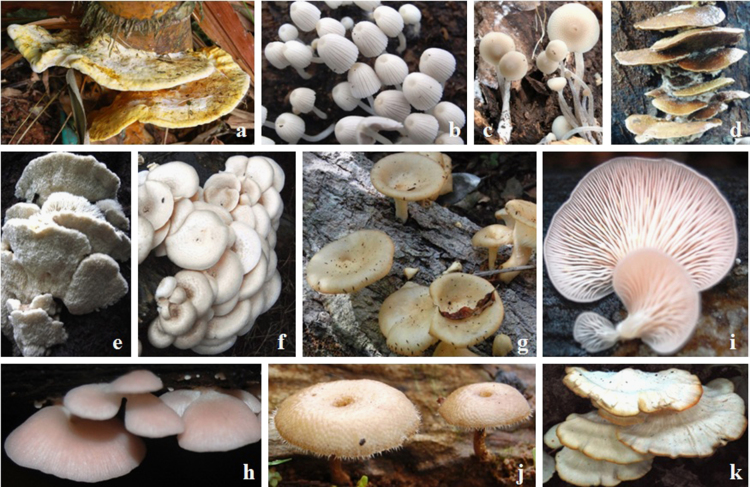
Selected mushrooms grown on wood/monocot stub: *Amylosporus campbellii* (a); *Coprinus disseminatoides* (b), *Filoboletus manipularis* (c), *Gyrodontium sacchari* (d), *Hericium cirrhatum* (e), *Lentinus squarrosulus* (f), *Pleurotus cornucopiae* (g), *P. eöus* (h, i), *Polyporus arcularius* (j) and *Royoporus spathulatus* (k).

**Fig. 2:**
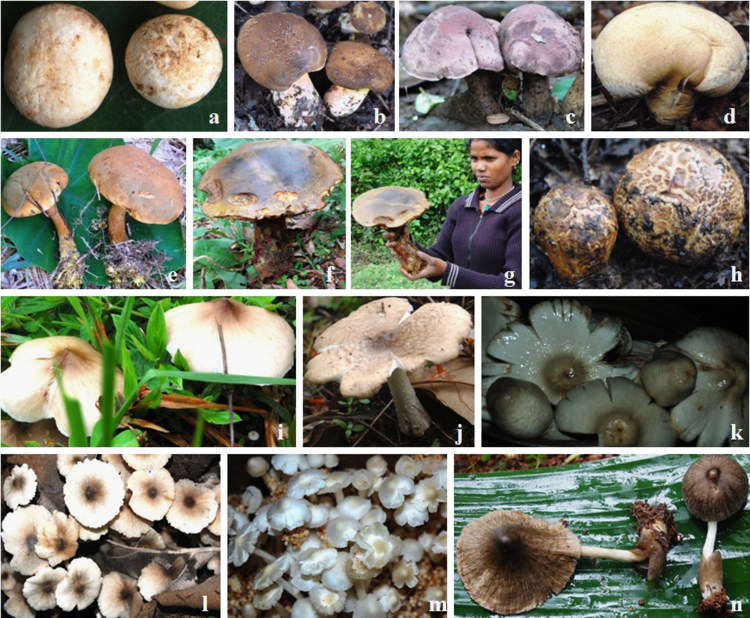
Selected mushrooms grown on soil: *Astraeus hygrometricus* (a), *Boletinellus merulioides* (b), *Boletus edulis* (c), *Lycoperdon utriforme* (d), *Phlebopus marginatus* (e), *P. portentosus* (f), Yerava tribe lady holding *P. portentosus* (g), *Scleroderma citrinum* (h), *Termitomyces clypeatus* (i), *T. fuliginosus* (j), *T. heimii* (k), *T. indicus* (l), *T. microcarpus* (m) and *Volvariella bombycina* (n).

Edible mushrooms constitutes alternative source of food against plant- or animal-derived food sources [1]. Despite cultivation of a few mushrooms in large scale [2], wild edible mushrooms are the major concern in food security of ethnic and tribal population [1]. Other than nutritional value, wild mushrooms are also contributed significantly as nutraceuticals [3]. The Western Ghats region of India constitutes a major hotspot of diversity of wild mushrooms [4]. Indigenous techniques of identification of habitats, edible mushrooms, substrate preference, collection and processing are yet to be explicitly documented. Table 1 composed of specific questionnaire adapted to document mainly the habitats, ethnic names, substrates, methods of processing (e.g. cooking, fried in oil, partially burn and pickling/salting), extent of occurrence and extent of edibility of wild mushrooms by the ethnic groups. To avoid overlap, separate questionnaire was used for each mushroom. Table 2, Table 3 possess systematic names, ethnic names, substrate preference, extent of occurrence and extent of edibility on wood/monocot stub and soils, respectively. Ethnic name of mushroom mainly dependent on the substrate (wood/monocot stub or soil), macromorphology (shape, size, color, difference in umbo and aroma/fragrance) and the names are often overlapping. For instance, the ethnic name is same for different mushrooms growing on a specific type of soil (*Phlebopus* spp. and *Termitomyces* spp.). The name differs for a specific mushroom when it grows on different type of wood/monocot stub (*Fistulina* spp. and *Pleurotus* spp.). Fig. 1, Fig. 2 consist of photographs of selected wild mushrooms of interest to ethnic groups grown on wood/monocot stub and soil, respectively. In addition to 51 wild edible mushrooms documented in this paper, several others are also recognized by the ethnic groups/tribes by different strategies (e.g. *Agaricus* spp., *Chlorophyllum* spp., *Lactarius* spp., *Lycoperdon* spp., *Macrolepiota* spp., *Russula* spp. and *Volvariella* spp.). This reveals the potentiality of indigenous knowledge of ethnic groups/tribes and further insights are necessary to document such aspects explicitly for future benefits.

**Table 1:** Questionnaire used for evaluation of edible mushrooms by ethnic groups/tribals in the Western Ghats region of India (separate questionnaire was used for each mushroom).

Date and season of survey:	?
Region of survey:	?
Name of ethnic group / tribe:	?
Vernacular name of mushroom:	?
Habitat of mushroom:	?
Substrate of mushroom:	Single / Multiple
Extent of occurrence:	Rare, frequent and most frequent
Up to what extent the mushroom is preferred?	Less, moderate and high
Any special consideration for identification?	?
Does the mushroom occur seasonally or in off season?	?
Does the mushroom appear in same place often?	?
Any specific effort for grow mushroom *ex situ*?	?
Any specific method for sampling and transport?	?
Which method followed for processing?	?
Any medicinal use of mushroom?	?
Any peculiarities of mushroom or habitat?	e.g. Mutualistic and canopy
What is the marketing strategy for financial benefits?	?

**Table 2:** Wild edible mushrooms of the Western Ghats region of India eaten by ethnic groups/tribals grown mainly on wood/monocot stub with their vernacular name (in parenthesis), substrates supported, extent of occurrence and edibility (in parenthesis) (occurrence: + rare, ++ frequent, +++ most frequent; edibility preference: *less, **moderate, ***high).

Mushroom	Substrate and vernacular name of mushroom	Number of substrates	Occurrence (and edibility)
*Amylosporus campbellii* (Berk.) Ryvarden (Fig. 1a)	On nodal region of degrading *Bambusa bambos* (Punda kum)	1	++ (*)
*Auricularia auricula-judae* (Bull.) Quél	Dead logs of *Artocarpus heterophyllus* (Chekke mara kum), *Bambusa bambos* (Punda kum), *Canarium strictum* (Dhoopa mara kum), *Cocos nucifera* (Tenge mara kum), *Dysoxylum malabaricum* (Devadaru mara kum) and *Mangifera indica* (Mange mara kum); Common ethnic name irrespective of substrate: Kemira-madri kum	6	+++ (*)
*Clitocybe infundibuliformis* (Schaeff.) Quél	Dead logs and exoperidium of fruits of *Cocos nucifera* (Tenge mara kum)	1	++ (***)
*Coprinus disseminatoides* Küher (Fig. 1b)	Stub of *Musa paradisiaca* (Bale mara kum)	1	++ (*)
*Filoboletus manipularis* (Berk.) Singer (Fig. 1c)	Dead wood and logs of *Madhuca neriifolia* (Nani mara kum / Toot kum).	1	+ (*)
*Fistulina hepatica* (Speg.) J.E. Wright	Trunk and tree holes of *Apodytes dimidiata* (Slate mathi mara kum / Kari mara kum), *Artocarpus hirsutus* (Ajine mara kum)*, Diospyros candolleana* (Kari mara kum)*, Elaeocarpus munroii* (Idinji mara kum)*, Memecylon umbellatum* (Udthale mara kum) *Salix tetrasperma* (Bayya mara kum) and *Syzygium cumini* (Nerale mara kum)*,.*	7	+ (***)
*Gyrodontium sacchari* (Spreng.) Hjortstam (Fig. 1d)	Dead standing stub of *Caryota urens* (Byne / Pane mara kum)	1	+ (*)
*Hericium cirrhatum* (Pers.) Nikol. (Fig. 1e)	Trunk and tree holes of *Euodia lunu-ankenda* (Chotva mara kum / Jool kum / Karadi kum)	1	+ (*)
*Lentinus patulus* Lév.	Dead logs of *Mangifera indica* (Mange mara kum)	1	++ (*)
*L. sajor-caju* (Fr.) Fr.	Dead logs of *Mangifera indica* (Mange mara kum)	1	+ (***)
*L. squarrosulus* Mont. (Fig. 1f)	Dead logs of *Artocarpus heterophyllus* (Chekke mara kum)*, Mangifera indica* (Mange mara kum) and *Anacardium occidentale* (Geru mara alambu).	3	+++ (*)
*Pleurotus cornucopiae* (Paulet) Rolland (Fig. 1g)	Dead logs of *Canarium strictum* (Dhoopa mara kum)	1	+ (***)
*P. djamor* (Rumph. ex Fr.) Boedijn	Dead logs and wood of *Artocarpus heterophyllus* (Chekke mara kum)*, Bombax ceiba* (Booruga mara kum)*, Caryota urens* (Byne / Pane mara kum)*, Cocos nucifera* (Tenga mara kum), *Erythrina subumbrans* (muruku mara kum)*,* dead stem of *Jatropha curcas* (Kachi kum), *Schefflera racemosa,* (Beth mara kum) and *Vernonia arborea* (Pookutti mara kum) and *Elaeis guineensis* (Thale mara kum)	9	+++ (***)
*P. eöus* (Berk.) Sacc. (Fig. 1h, i)	Dead logs of *Bombax ceiba* (Booruga mara kum)*, Caryota urens* (Byne / Pane mara kum) and *Cocos nucifera* (Tenga mara kum) *Elaeis guineensis* (Thale mara kum)	4	+ (***)
*P. flabellatus* Sacc.	Dead logs of *Artocarpus heterophyllus* (Chekke mara kum)*, Diospyros candolleana* (Kari mara kum)*, Ficus amplissima* (Bairi mara kum)*, Ficus drupacea* (Goli mara kum)*, Ficus racemosa* (Athi mara kum)*, Michelia champaca* (Sampige mara kum), *Schefflera racemosa* (Beth mara kum) and *Vernonia arborea* (Pookutti mara kum).	8	+++ (***)
*P. ostreatus* (Jacq.) P. Kumm.	Dead logs of *Cinnamomum malabatrum* (Nerange mara kum) *Elaeocarpus serratus* (Athakoome mara kum) and *Persea macrantha* (Kulumavu mara kum)*,*	3	++ (***)
*Polyporus arcularius* Lázaro Ibiza (Fig. 1j)	Dead wood and logs of *Madhuca neriifolia* (Nani mara kum)	1	+ (**)
*Royoporus spathulatus* (Jungh.) A.B. De (Fig. 1k)	Dead stub of *Caryota urens* (Byne / Pane mara kum)	1	+++ (**)

**Table 3:** Wild edible mushrooms of the Western Ghats region of India eaten by ethnic groups/tribals grown mainly on soil with their vernacular name (in parenthesis), substrate supported, extent of occurrence and edibility (in parenthesis) (mutualistic association: ^Ψ^ectomycorrhizal, ^ω^termites; occurrence: + rare, ++ frequent, +++ most frequent; edibility preference: *less, **moderate, ***high).

Mushroom	Substrate and ethnic name of mushroom	Number of substrates	Occurrence (and edibility)
^Ψ^*Astraeus hygrometricus* (Jungh.) A.B. De (Fig. 2a)	Partially submerged in soil (Kallu alambu / Kall anabe)	1	+++ (***)
^Ψ^*Austroboletus gracilis* (Peck) Wolfe	On soil (Chalae pandi kum)	1	+ (**)
^Ψ^*Boletinellus merulioides* (Peck) Wolfe (Fig. 2b)	On Soil under *Mangifera indica*, *Artocarpus heterophyllus* and *Cassine glauca* (Manja pandi kum)	3	++ (**)
^Ψ^*Boletus edulis* Bull. (Fig. 2c)	On soil under *Hydnocarpus pentandra, Holigarna nigra* and *Canarium strictum* (Chalae pandi kum)	3	++ (**)
^Ψ^*B. reticulatus* Schaeff.	On lateritic soil (Pandi kum)	1	+ (**)
*Gymnopilus junonius* (Fr.) P.D. Orton	On burnt soil with burnt *Bambosa bamobs* debris (Taen kum)	1	++ (*)
^Ψ^*Lycoperdon utriforme* Bull. (Fig. 2d)	On soil under *Acacia mangium* and *Artocarpus heterophyllus* (Buguri / Urutu / Goomatte kum)	2	+++ (*)
^Ψ^*Phlebopus marginatus* Watling & N.M. Greg. (Fig. 2e)	On soil under *Bamboo burmanica* thicket (Punda pandi kum)	1	+ (*)
^Ψ^*P. portentosus* (Berk. & Broome) Boedijn (Fig. 2f, g)	On soil under *Coffea robusta* (Mannu pandi kum / Kapi pandi kum)	1	++ (**)
^Ψ^*Rubinoboletus caespitosus* T.H. Li & Watling	On forest soil (Chalae pandi kum)	1	+ (**)
^Ψ^*Scleroderma citrinum* Pers. (Fig. 2h)	On soil under *Artocarpus heterophyllus*, *Dysoxylum malabaricum* and *Schefflera racemosa* (Kadya kum)	3	+ (***)
^Ψ^*Suillus brevipes* (Peck) Kuntze	On forest soil (Pandi kum)	1	++ (**)
^Ψ^*S. placidus* Singer	On forest soil (Pandi kum)	1	++ (**)
^Ψ^*S. tomentosus* Singer	On forest soil (Pandi kum)	1	++ (**)
^ω^*Termitomyces clypeatus* R. Heim (Fig. 2i)	Soil with termites (Nethale kum)	1	+++ (***)
^ω^*T. eurrhizus* (Berk.) R. Heim	Soil with termites (Gonnae kodae kum)	1	+++ (***)
^ω^*T. fuliginosus* R. Heim (Fig. 2j)	Soil with termites (Kundu kodae kum)	1	++ (***)
^ω^*T. globulus* R. Heim & Gooss.-Font.	Soil with termites (Urutu kodae kum / Aeri kodae kum)	1	++ (***)
^ω^*T. heimii* Natarajan (Fig. 2k)	Soil with termites (Alandi kum / Heggala alambu / Heggala anabae)	1	++ (***)
^ω^*T. indicus* Natarajan (Fig. 2l)	Soil with termites (Nuchi kum)	1	++ (***)
^ω^*T. lanatus* R. Heim	Soil with termites (Joolu kodae kum / Karadi kodae kum)	1	+(***)
^ω^*T. le-testui* (Pat.) R. Heim	Soil with termites (Gantae kodae kum)	1	+ (***)
^ω^*T. mammiformis* R. Heim	Soil with termites (Amme kodae kum)	1	+ (***)
^ω^*T. medius* R. Heim & Grassé	Soil with termites (Pill kodae kum)	1	++ (***)
^ω^*T. microcarpus* (Berk. & Broome) R. Heim (Fig. 2m)	On termite faecal pellets associated with soil (Katola kum / Akki kum) and leaf litter with termite faecal pellets (Kokkalae kum)	2	+++ (***)
^ω^*T. robustus* (Beeli) R. Heim	Soil with termites (Tholu / Nar kodae kum)	1	++ (***)
^ω^*T. schimperi* (Pat.) R. Heim	Soil with termites (Baeru alambu / Baeru anabe / Beeru kodae kum)	1	+ (***)
^ω^*T. spiniformis* R. Heim	Soil with termites (Nai nethale kum)	1	+ (***)
^ω^*T. striatus* (Beeli) R. Heim	Soil with termites (Pullae / Puthu kum)	1	+++ (***)
^ω^*T. titanicus* Pegler & Ouearce	Soil with termites (Baari balliya kodae kum / Balliya punda kodae kum)	1	+ (***)
^ω^*T. tylerianus* Otieno	Soil with termites (Kaad kodae kum)	1	++ (***)
^ω^*T. umkowaan* (Cooke & Massee) D.A. Reid	Soil with termites (Nai kodae alambu)	1	++ (***)
*Volvariella bombycina* (Schaeff.) Singer (Fig. 2n)	On soil with degrading banana (*Musa paradisiaca*) (Baale kum)	1	+ (*)

## 2 Methods of survey, data collection and analysis

### 2.1 Geographic locations and ethnic groups

Several locations mainly in Kodagu region of the Western Ghats (11°56', 12°52' N and 75°22', 76°11' E) were surveyed for edible wild mushrooms based on the guidance of ethnic groups/tribals during the monsoon and post-monsoon seasons of 2012–2016. Locations surveyed includes forest reserves, sacred groves, shola forests, lateritic scrub jungles, coffee agroforests, orchards, plantations (*Acacia, Anacardium, Areca* and *Musa*), bamboo thickets, grass lands and bunds of paddy fields. In addition, residential locations such as kitchen gardens, wastelands, termite-infested regions and cattle sheds were also locations of survey. A wide range of ethnic groups have been consulted include: Amma-Kodava, Are-Gowda, Billava, Iri, Jamma-Mapale, Kembatti-Poliya, Kodava, Kukka-Poliya, Kudiya, Kuraba, Madiyala, Meda, Okkaliga-Gowda, Panika, Patta, Peggade and Yerava.

### 2.2 Questionnaire

We compiled the data on the ethnic knowledge acquired on edible mushrooms in different habitats, substrates and tree species based on specific questionnaire (Table 1). The ethnic names of mushrooms are dependent on the habitat and tribes inhabiting in specific region (forest, grassland and orchard), supporting woody (and monocot stub) substrate (logs, bark, twigs, shells of nuts and roots as ectomycorrhizal), local name of tree species (dead part of tree and tree holes), soils (termite mound, humus, bunds of paddy fields), growth of mushroom (overall nature of group of mushroom), extent of mushroom biomass (rare, moderate and profuse) and extent of edibility (low, moderate and high). Name of each mushroom has a suffix 'kum' (= mushroom), if it grows on a specific wood/monocot stub of a tree/plant species, it has prefix of specific vernacular name of tree followed by 'mara' (= tree). Several wild mushrooms are marketed by ethnic groups/tribes for their livelihood. Those wild mushrooms available in great biomass especially in lateritic soils (e.g. *Astraeus hygrometricus*) and termite soils (e.g.*, Termitomyces clypeatus, T. heimii, T. indicus, T. schimperi, T. striatus* and *T. umkowaan*), large enough to handle and stay for considerable duration are preferred for marketing. Such marketed mushrooms costs up to Rupees 500–600 per kg (∼U$ 8–9/kg).

### 2.3 Ecological groups

Based on ethnic knowledge of 17 tribals of Kodagu region of the Western Ghats, we visited 10 habitats, evaluated wood/monocot stub of about 30 plant species and screened about 10 soils types (Table 2, Table 3). Edible mushrooms of the Western Ghats sampled in this survey have been broadly classified into two major ecological groups: a) wood-/monocot stub-dependent mushrooms; b) soil-dependent mushrooms. Further, soil-dependent mushrooms could be sub-divided into: a) different soils; b) ectomycorrhizal with roots; c) association with termites. The quantity as well as quality of wood/monocot stub materials differed in different locations surveyed. There are several varieties of wood/hard materials (e.g. softwood, hardwood, bamboo and monocot stub) and their niches (fallen, standing dead, bark, tree holes, canopy and roots). Similarly, the quality of soil also varies in different locations assessed (e.g. humus, termite mound, termite-infested soil, lateritic soil and soil embedded with leaf litter/wood pieces/shredded wood/wood powder). Such differences in substrates have major influence on extent of occurrence (biomass), distribution and diversity of wild mushrooms. Human interferences have negative impact on the occurrence and diversity of wild mushrooms especially tampering organic matter (deforestation, removal or extraction of wood and extensive pruning of tree canopies in agroforests) and other actions which enhances soil erosion (clearing vegetation, leveling the terrain, destruction of termite mounds and transformation of grasslands) have direct or indirect impact on the diversity, distribution and biomass of wild mushrooms.

Among 33 wild mushrooms occurred on wood/monocot stub, *Pleurotus djamar* occurred on the highest number of materials (9), followed by *Pleurotus flabellatus* (8), *Fistulina hepatica* (7), *Auricularia auricula-judae* (6) and the rest of the species occurred on 1–4 wood/monocot stubs (Table 2). Substrate choices of soil-dependent mushrooms are narrow and occurred between one and three types of soil (Table 3). However, such narrow specificity revealed two major niches of mushrooms like ectomycorrhizal with specific tree species (1–3) and termite mound/termite infested soil (1).

Ethnic groups and tribals are aware that some edible wild mushrooms erupt suddenly. For instance, *Fistulina hepatica* occurs during untimely rains especially in tree holes (post-monsoon: November–December or summer: March–April). They also aware that some hallucinogenic mushrooms grow on degrading grass and herbivore dung (e.g. *Coprinus* spp. and *Psylocybe* spp.). Almost all wild mushrooms are consumed in tender stage, ethnic groups/tribals are aware of availability of tender wild mushrooms in a specific location, substrate and season. Some people need first-aid or to be hospitalized for treatment as they consume unknowingly the mature instead of tender mushrooms. For example, from tender to mature, the gills of *Agaricus* spp. turn into purple; the white flesh of *Astraeus* spp., *Scleroderma* spp. and *Lycoperdon* spp. turns into spores. If the time lapse between collection and consumption is wide, which may leading to maturity of some mushrooms thus becoming unfit for consumption.

### 2.4 Analysis

The number of geographic locations visited, tribes consulted, wood/monocot plant species screened and substrates surveyed have been documented. The wild mushrooms are identified based on the specific ethnic/traditional knowledge: a) Presence of insects and larvae (*Drosophila*, ants, housefly and maggots); b) Typical strong almond-like aroma for all edible wild mushrooms (sensed by a few ethnic people or tribals); c) Macromorphology (shape, size, colour, surface features, shape of umbo and presence of pseudorhzae), habitat and substrate also decides edibility (e.g. *Fistulina*, *Pleurotus* and *Termitomyces*); d) Mutualistic associations with plants (ectomycorrhizae) or animals (termites) (e.g. *Astraeus, Scleroderma* and *Termitomyces*); e) Change in colour of gills or endoperidial flesh (white to coloured) will decide its edibility (usually such change in colour due to maturation is associated with food poisoning or indicates non-edibility).

### 2.5 Glossary for ethnic names

Glossary for vernacular language has been prepared and documented here. Many names of mushrooms occurring on specific wood/monocot stub materials has first name as tree name followed by 'mara' (= tree) and 'kum' (= mushroom) (Table 2). Those occurring on soils also have different ethnic names (Table 3). Following are commonly used vernacular terms for different mushrooms: Akki = Rice, Alambu = Mushroom, Amme = Breast-like, Anabe = Mushroom, Baari = Big, Balliya = Big, Baeru = Root, Beeru = Root, Buguri = Top / Pedestal, Chalae = Purple, Gante = Bell, Gonnae = Slime, Goomatte = Doom-like, Joolu = Drooping hairs, Kaad = Forest, Kall = Stone, Kapi = Coffee, Karadi = Bear, Kodae = Umbrella, Kokkalae = Leaf litter, Kum = Mushroom, Kundu = Holes, Mara = Tree, Manja = Yellow, Mannu = Soil, Nai = Too many, Nar = Fibre, Nethale = Sharp umbo, Nuchi = Rice grits, Pandi = Pig, Pill = Grass, Punda = Bamboo, Taen = Honey, Tholu = Skin, Urutu = Round. However, some ethnic terms have no specific or clear meaning but used conventionally to designate some mushrooms (e.g. Alandi, Heggala, Kadya and Katola).

## Conflict of Interest

There is no conflicts of interest to publish this research paper
